# The dual role of T cells in solid organ transplant rejection and immune tolerance

**DOI:** 10.3389/fimmu.2026.1787579

**Published:** 2026-03-26

**Authors:** Zixuan Zhang, Nian Zhao, Yanxia Miao, Yubao Sun, Chen Liu, Ning Cao, Chunping Liu, Yunlong Ding, Jun Li, Niannian Li

**Affiliations:** 1 School of Clinical Medicine, Shandong Second Medical University, Weifang, Shandong, China; 2 Weifang People’s Hospital, Weifang, Shandong, China

**Keywords:** CD4+ T cells, CD8+ T cells, immune rejection, immune tolerance, solid organ transplantation, T cells, Tregs

## Abstract

Solid organ transplantation, a major breakthrough in modern medicine, has saved countless patients with end-stage organ failure. However, immune rejection remains the primary obstacle to transplant success. As the central effector cells of adaptive immunity, T cells drive acute rejection by directly recognizing donor alloantigens (such as MHC molecules) or indirectly recognizing processed donor antigens presented by antigen-presenting cells. Recent studies have revealed the dual roles of distinct T cell subsets in rejection or tolerance: pro-inflammatory Th1, Th2, Th17, and CD8^+^ cytotoxic T cells(CTLs) exacerbate tissue damage by secreting cytokines such as IFN-γ and IL-17, whereas regulatory T cells(Tregs) promote graft tolerance by suppressing effector T cell activation and maintaining immune homeostasis. This article systematically reviews the molecular mechanisms of T cell-mediated rejection, functional heterogeneity among T cell subsets, and their differential impacts on various types of solid organ transplants.

## Introduction

**Figure 1:**
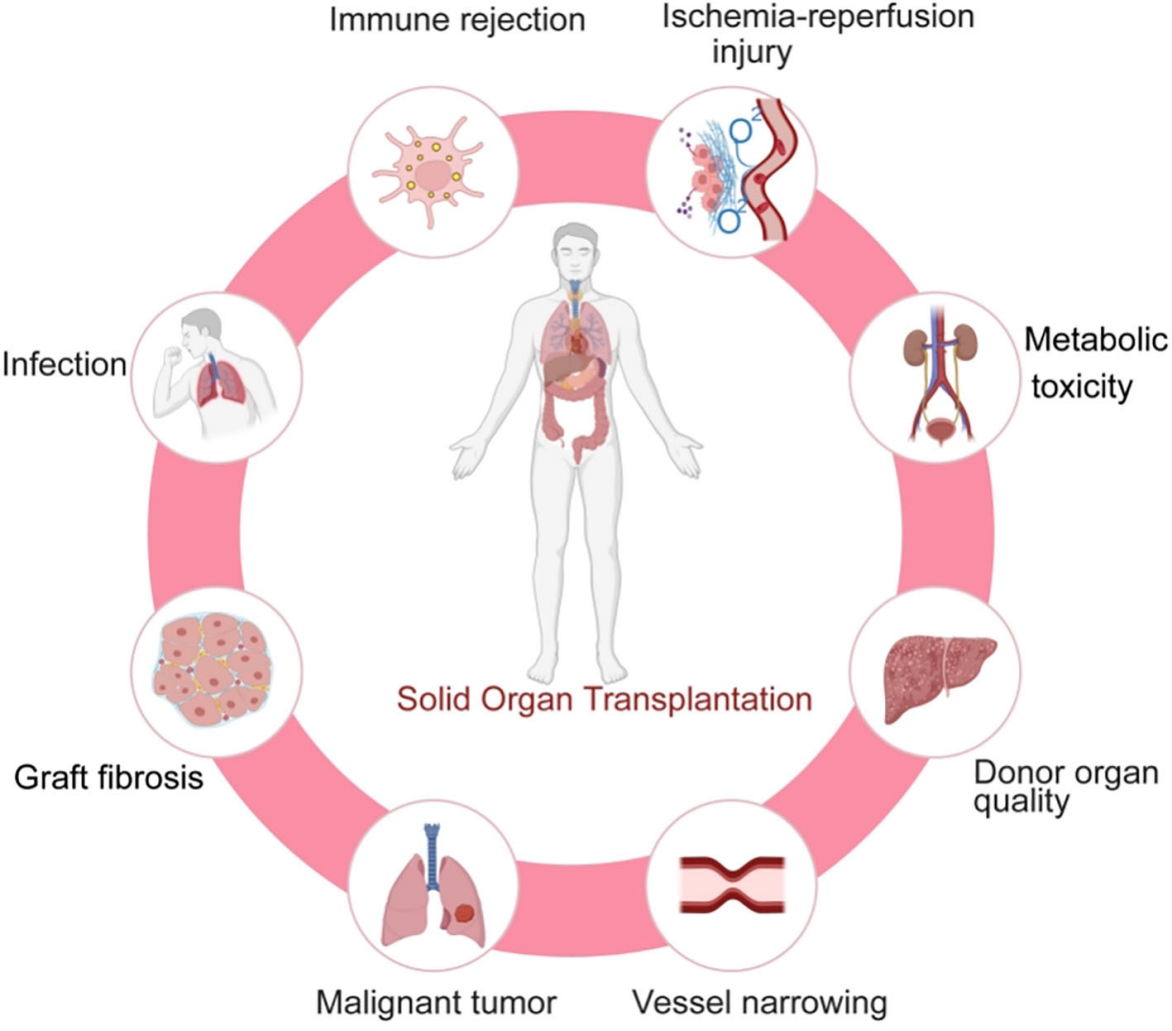
The challenges faced by solid organ transplantation.

Solid organ transplantation serves as an effective treatment for end-stage organ failure, significantly improving patients’ quality of life. Certain congenital diseases can also be cured through transplantation, while for some organ-confined malignancies, the procedure achieves dual therapeutic goals by simultaneously removing both the diseased organ and tumor (1). Since the first successful kidney transplantation in the mid-20th century (2), the technique has been extensively applied to multiple organs, including heart, liver, lung, kidney, intestine, and pancreas, with remarkable advancements. Nevertheless, long-term graft survival remains challenging due to multiple factors, including immune rejection, drug side effects (3), donor organ quality, and postoperative complications (4), among which immune rejection constitutes the primary limiting factor (**Figure 1**) (2).

**Figure 2:**
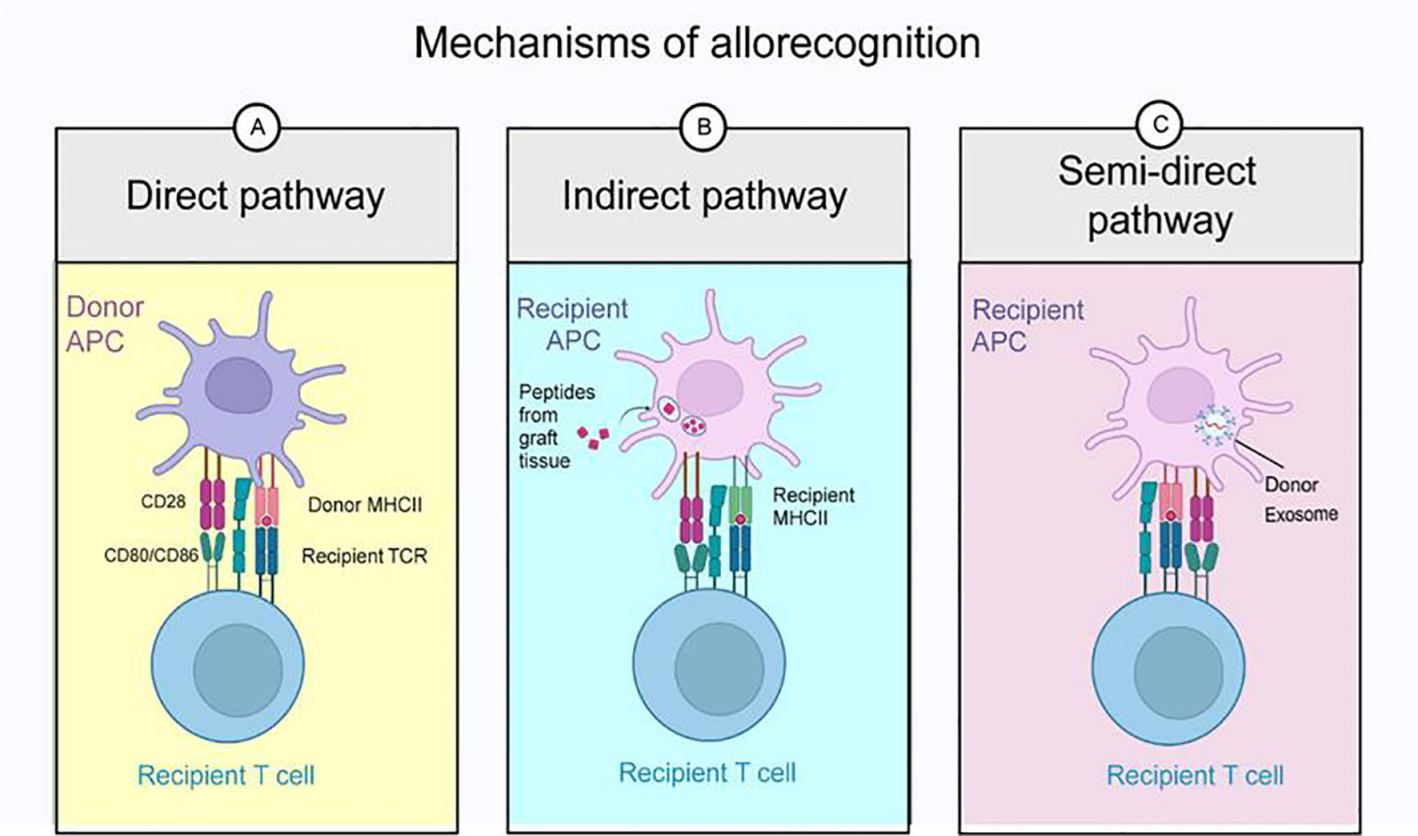
Mechanisms of allorecognition. After allogeneic transplantation, recipient T cells recognize donor antigens through direct pathways, indirect pathways, and semi-direct pathways. **(A)** Direct pathway: Recipient T cells directly bind to MHC molecules on the surface of donor APCs via their TCRs. **(B)** Indirect pathway: Donor antigens are taken up, processed, and presented by recipient antigen-presenting cells(APCs) to recipient T cells. **(C)** Semi-direct pathway: Donor MHC molecules are acquired by host APCs through mechanisms such as intercellular transfer or exosomes, followed by cross-presentation to further activate T cell responses.

In transplant immunity, T cells function as the principal effector cells orchestrating graft rejection through multifaceted mechanisms of alloantigen recognition and attack, thereby driving both acute and chronic rejection processes (5). Elucidating the molecular and cellular basis of T cell-mediated rejection is fundamental to developing next-generation immunosuppressive therapies and enhancing long-term graft survival. After allogeneic transplantation, recipient T cells recognize donor antigens through three distinct pathways: direct pathway, indirect pathway, and semi-direct pathway (**Figure 2**). These recognition pathways orchestrate T cell activation, clonal expansion, and effector differentiation, ultimately culminating in graft injury through both cellular and humoral mechanisms.

Activated T cells differentiate into functionally distinct effector subsets, each exhibiting specialized immunological functions. Under the influence of the local microenvironment, activated CD4^+^ T cells can further differentiate into a variety of effector subsets, including Th1, Th2, Th17, and Th22, among others. These subsets play complex roles in rejection by secreting distinct cytokine profiles. For example, Th1 cells secrete IFN-γ and TNF-α, activating macrophages and enhancing inflammatory responses, while Th17 cells promote neutrophil infiltration and tissue fibrosis through IL-17 (6, 7). CD8^+^ CTLs directly recognize allogeneic MHC class I molecules on graft cell surfaces, inducing target cell apoptosis through perforin and granzyme release (8). Furthermore, the persistence of memory T cells(Tm) enables more rapid and potent rejection responses, which may ultimately lead to graft failure even under long-term immunosuppressive therapy (9).

Tregs maintain immune tolerance by suppressing the activation and proliferation of effector T cells (10). However, under inflammatory conditions or inadequate immunosuppression, Treg function may be impaired, and a subset of Tregs can even convert into pro-inflammatory effector cells, exacerbating rejection (11). Thus, modulating the balance between Tregs and effector T cells has become a critical focus in transplant immunology research.

This review aims to systematically elucidate the key mechanisms and pathways through which T cells mediate transplant rejection, thoroughly investigate the interactions between effector T cells and Tregs in immune balance along with the impact of their functional dysregulation, analyze the limitations of current therapies and provide theoretical foundations for developing targeted anti-rejection strategies. Ultimately, it prospects future research directions focused on achieving long-term transplant immune tolerance through precise regulation of T cell function.

### T cells synergize with both innate and adaptive immune systems

**Figure 3:**
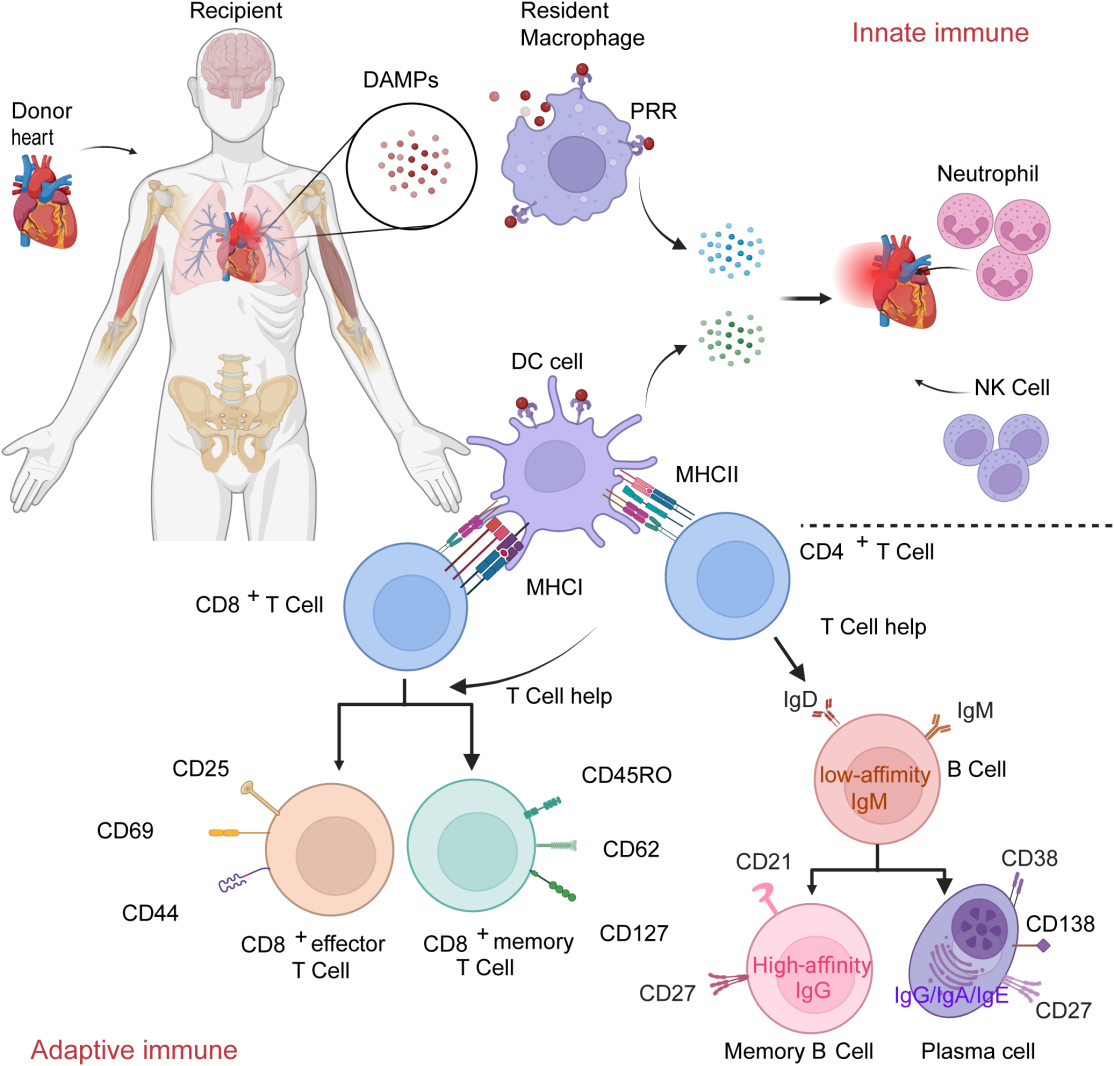
The processes of innate immunity and adaptive immunity. Taking heart transplantation as an example, during the transplantation process, factors such as ischemia and surgical injury to the donor organ lead to the substantial release of DAMPs. DAMPs bind to PRRs on macrophages and dendritic cells, activating the innate immune system. This triggers the secretion of pro-inflammatory cytokines, initiates an inflammatory response, and recruits neutrophils and natural killer(NK) cells to the heart. TCRs recognize the MHC-donor antigen peptide complexes on the surface of dendritic cells. Simultaneously, the highly expressed CD80/CD86 molecules on dendritic cells bind to CD28 on the surface of T cells. Driven by these dual signals and cytokines, naïve T cells become fully activated, undergo clonal proliferation, and differentiate into effector T cells—including cytotoxic CD8^+^ T cells and helper CD4^+^ T cells—thereby initiating acute rejection. In the later stage of the primary immune response, a portion of the effector cells transforms into long-lived memory lymphocytes. Memory T cells can rapidly mount a stronger response upon re-encountering the same antigen. CD4^+^ T cells promote B cells to undergo affinity maturation and class switching in the germinal centers, differentiating into memory B cells and long-lived antibody-secreting plasma cells, thus establishing antibody-mediated immune memory. The antibodies secreted by these cells are present in bodily fluids, enabling rapid recognition and neutralization of reinvading pathogens or antigens.

Immune rejection occurs when a donor organ is transplanted into a recipient, and the recipient’s immune system recognizes the graft as foreign, triggering a series of immune responses to attack it. This process primarily involves the synergistic action of innate and adaptive immunity (6). (**Figure 3**).

The innate immune response helps initiate and shape adaptive immune responses mediated by T cells and B cells against the donor organ in the context of transplantation. In the simplified three-signal model, the first signal for T cell activation is provided by TCR recognition of antigen; the second signal is co-stimulation provided by APCs; the third signal derives from inflammatory cytokines produced upon innate immune activation, which may act directly on T cells and/or indirectly by upregulating costimulatory molecules on APCs. Effector T cell infiltration into grafts triggers rejection, establishing a bidirectional regulation that achieves both rapid anti-infection responses and specific immune memory (7). Upon pathogen detection via pattern recognition receptors(PRRs), APCs upregulate costimulatory molecules and secrete cytokines that determine naive T cell differentiation into distinct effector subsets. Activated T cells then modulate innate immune responses through cytokine feedback. For example, in transplantation, donor or recipient dendritic cells(DC) present donor antigens directly and express high levels of CD80/CD86, thereby effectively activating host alloreactive T cells (12), and secrete IL-6 and TNF-α (13). These activated alloreactive T cells subsequently feedback regulation of innate immune responses (14).

The adaptive immune system is primarily orchestrated by T lymphocytes and B lymphocytes, with T cells occupying a central and pivotal position within this network (15, 16), which is manifested in antigen-specific recognition, immune memory, diversity of effector functions, and immunoregulation (17, 18). T cells precisely recognize peptide-MHC complexes through TCRs to initiate antigen-specific immune responses (19, 20), and form memory T cells after primary immunization to provide long-term immune protection. In the process of immune defense against a graft, the initially activated innate immune response is characterized by rapid but non-specific recognition of donor antigens; in contrast, the subsequently activated adaptive immune response generates highly specific and durable immune protection through clonal expansion and memory cell differentiation (21, 22), which forms the critical basis for the occurrence of both acute and chronic rejection.

### T cells and classification of immune rejection

Hyperacute rejection is primarily driven by antibodies and complement, yet T cells can indirectly influence its occurrence by modulating B cell responses and the inflammatory microenvironment (23, 24). Acute rejection is one of the primary causes of graft function loss, yet it can generally be reversed through timely diagnosis and treatment. Recipient T cells are activated either by directly recognizing allogeneic MHC molecules on donor APCs or indirectly recognizing donor-derived antigen peptides presented by recipient APCs. Subsequently, they proliferate and differentiate under the influence of costimulatory signals such as CD28/B7, CD40/CD40L, and cytokines (25). Acute rejection is classified into antibody-mediated rejection(AMR) or acute cellular rejection(ACR). The occurrence of ACR is a T cell-mediated immune response, in which the recipient’s T cells recognize foreign antigens on the donor organ, become activated, and undergo extensive clonal expansion, and then lead to graft injury through two primary mechanisms: direct killing by CD8^+^ CTLs and inflammatory attacks orchestrated by CD4^+^ T cells. Th1 cells are the predominant effector subset in ACR, and the IFN-γ they secrete can activate macrophages, induce inflammatory infiltration, and promote the proliferation and cytotoxic function of CD8^+^ T cells. A deeper understanding of these immune recognition mechanisms may provide new therapeutic strategies to effectively reduce the incidence of ACR (26).

The pathological characteristics of chronic rejection are primarily manifested by vascular intimal hyperplasia, interstitial fibrosis, and progressive deterioration of graft function (27). Although current clinical interventions are effective in controlling acute rejection, complications from long-term immunosuppression and the irreversible nature of chronic rejection remain major challenges in the field of transplantation. Future research needs to further focus on the dynamic balance mechanism between effector and regulatory T cells.

### Dual-layer regulatory mechanisms of T cell-mediated immune tolerance

The outcome of organ transplantation hinges on the direction of the T cell immune response; these lymphocytes, screened by the central immune system and functioning within the peripheral immune system, ultimately determine whether the graft is accepted or attacked. The induction of immune tolerance can prevent T cell-mediated graft rejection and graft-versus-host disease(GVHD) (3). Therefore, the establishment of immune tolerance is critical for the long-term survival of transplanted organs (28).

Central tolerance primarily occurs in central immune organs such as the thymus and bone marrow. In the thymus, T lymphocytes undergo positive selection and negative selection during their development. Positive selection refers to the process where T lymphocytes interact via their TCR with MHC molecules presented by thymic epithelial cells. Only T cells capable of recognizing self-MHC molecules survive and proceed to further maturation, while those that fail to bind undergo apoptosis. This mechanism ensures that mature T lymphocytes are restricted to recognizing self-MHC, thereby preventing immune attacks against the body’s own tissues (29). Negative selection, on the other hand, refers to the elimination of T lymphocyte clones with high affinity for self-antigens in the thymus, thereby preventing the generation of autoreactive T cells.

**Figure 4:**
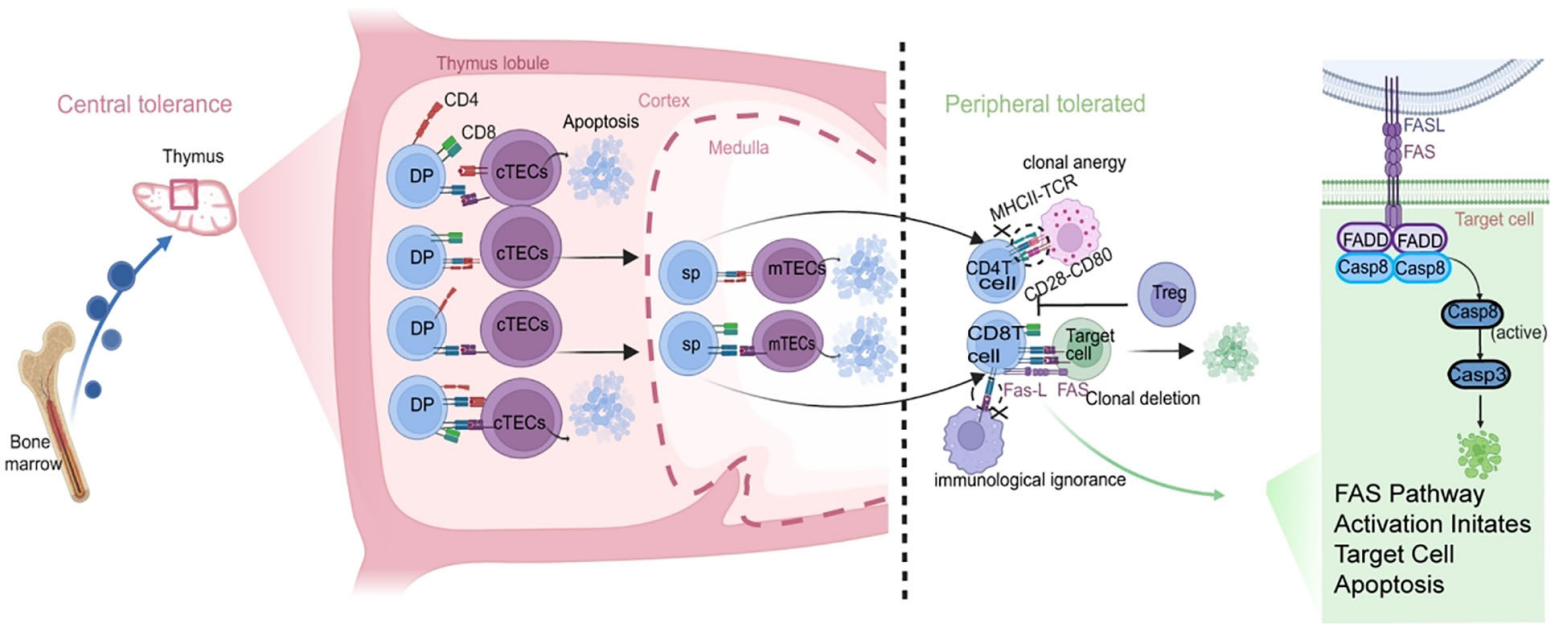
T cells in central and peripheral immunity. Positive selection occurs in the thymic cortex, where T lymphocytes undergo selection based on the interaction between their TCRs and MHC molecules expressed on thymic epithelial cells. T cells that successfully recognize self-MHC molecules survive and continue maturation, while those that fail to bind undergo apoptosis. Negative selection takes place in the thymic medulla, where T lymphocytes exhibiting high affinity for self-antigens are eliminated to prevent the generation of autoreactive T cells. However, some autoreactive T cells may escape central tolerance and migrate to peripheral lymphoid tissues. Mechanisms such as clonal anergy, clonal deletion, suppression by Tregs, and immune ignorance help reduce the number of autoreactive lymphocytes and suppress autoimmune responses. Clonal anergy, Treg-mediated active suppression, clonal deletion, and immunological ignorance can effectively reduce the number of autoreactive lymphocytes and inhibit the occurrence of immune rejection reactions. Clonal anergy: Upon antigen recognition, T cells enter a functionally quiescent state due to the absence of costimulatory signals, rendering them unable to proliferate or exert effector functions. Clonal deletion: T cells persistently activated by high antigen concentrations are physically eliminated through apoptosis. Treg-mediated active suppression: Tregs directly inhibit the activation and function of effector T cells through cell contact or the secretion of inhibitory factors. Immunological ignorance: Self-antigens, due to low concentration or sequestration, escape recognition by T cells, thereby preventing an immune response. Fas-mediated apoptotic pathway is a critical signaling cascade in programmed cell death. Upon binding of FasL to Fas, the DD recruits the adaptor protein FADD. FADD then binds to procaspase-8, inducing its autocleavage and conversion into active caspase-8. Activated caspase-8 can activate caspase-3, leading to apoptosis.

Central tolerance alone is insufficient to fully prevent immune rejection, as some autoreactive T lymphocytes may escape thymic deletion and migrate to peripheral immune organs. In such cases, peripheral tolerance mechanisms become critical for maintaining immune homeostasis (28–30). Clonal deletion helps reduce the number of donor-specific T cells, thereby limiting the occurrence of rejection (31, 32). Clonal anergy involves the two-signal requirement for T cell activation. In the context of organ transplantation, if donor antigens are presented by non-professional APCs or if costimulatory signals are blocked, T cells may receive antigen stimulation but lack the second signal, thereby entering a state of clonal anergy. Although such unresponsive T cells persist, they lose the ability to proliferate and produce effector cytokines, rendering them incapable of mounting an effective anti-graft immune response (29, 33). Tregs are a specialized subset of T lymphocytes with immunosuppressive functions that play a pivotal role in maintaining immune tolerance and homeostasis. Tregs primarily exert their suppressive effects through two mechanisms: direct cell-to-cell contact and secretion of inhibitory cytokines (such as IL-10 and TGF-β), which effectively suppress the activation and proliferation of effector T cells (34–37). In organ transplantation, Tregs can migrate to the graft site and actively inhibit the development of immune rejection responses. Furthermore, Tregs possess the capacity to modulate the function of other immune cells, including inhibiting dendritic cell maturation and antigen-presenting capacity, as well as reducing antibody production by B lymphocytes (29). Immunological ignorance refers to a state where T cells, despite possessing antigen specificity, remain unactivated due to low antigen concentration or restricted antigen presentation. This mechanism partially contributes to the immune privilege characteristics observed in transplanted grafts (**Figure 4**) (38, 39). Fas(CD95)-mediated apoptosis is a central signaling pathway of the extrinsic apoptotic cascade, functioning as a precise chain reaction akin to a “death switch.” The process initiates when Fas ligand(FasL) expressed on effector cells binds to Fas receptor on the surface of the target cell, triggering receptor trimerization (40). The pivotal step occurs on the inner side of the cell membrane: the death domains(DD) of the trimerized receptors recruit the adaptor protein Fas-associated death domain protein(FADD) (41), which in turn uses its death effector domains to recruit large quantities of inactive procaspase-8, collectively assembling the “Death-Inducing Signaling Complex”(DISC). Within the DISC, the highly concentrated procaspase-8 molecules undergo autocleavage, converting into the active initiator protease caspase-8. Activated caspase-8 then cleaves and activates the downstream executioner protease procaspase-3, transforming it into caspase-3 (42). As the ultimate executor of apoptosis, caspase-3 systematically cleaves hundreds of key intracellular substrates, such as disrupting DNA repair enzymes, degrading cytoskeletal proteins, and activating endonucleases, leading to DNA fragmentation (43, 44). This series of irreversible biochemical events ultimately manifests as the classic morphological hallmarks of apoptosis: cell shrinkage, chromatin condensation, membrane blebbing to form apoptotic bodies, which are subsequently cleared by phagocytes to prevent inflammatory responses (45).

The establishment of immune tolerance relies on a two-tiered precision regulatory system that operates from the central to the peripheral level. At the central level, the thymus eliminates self-reactive T cell clones at their source through positive and negative selection mechanisms, ensuring the export of mature T lymphocytes that are non-responsive to self-antigens. At the peripheral level, the coordinated action of multiple complementary mechanisms continuously monitors and suppresses self-reactive or alloreactive T cells that may have escaped to the periphery, thereby maintaining immune homeostasis.

### The pro-rejection effects of CD4^+^ T cell effector subsets

**Figure 5:**
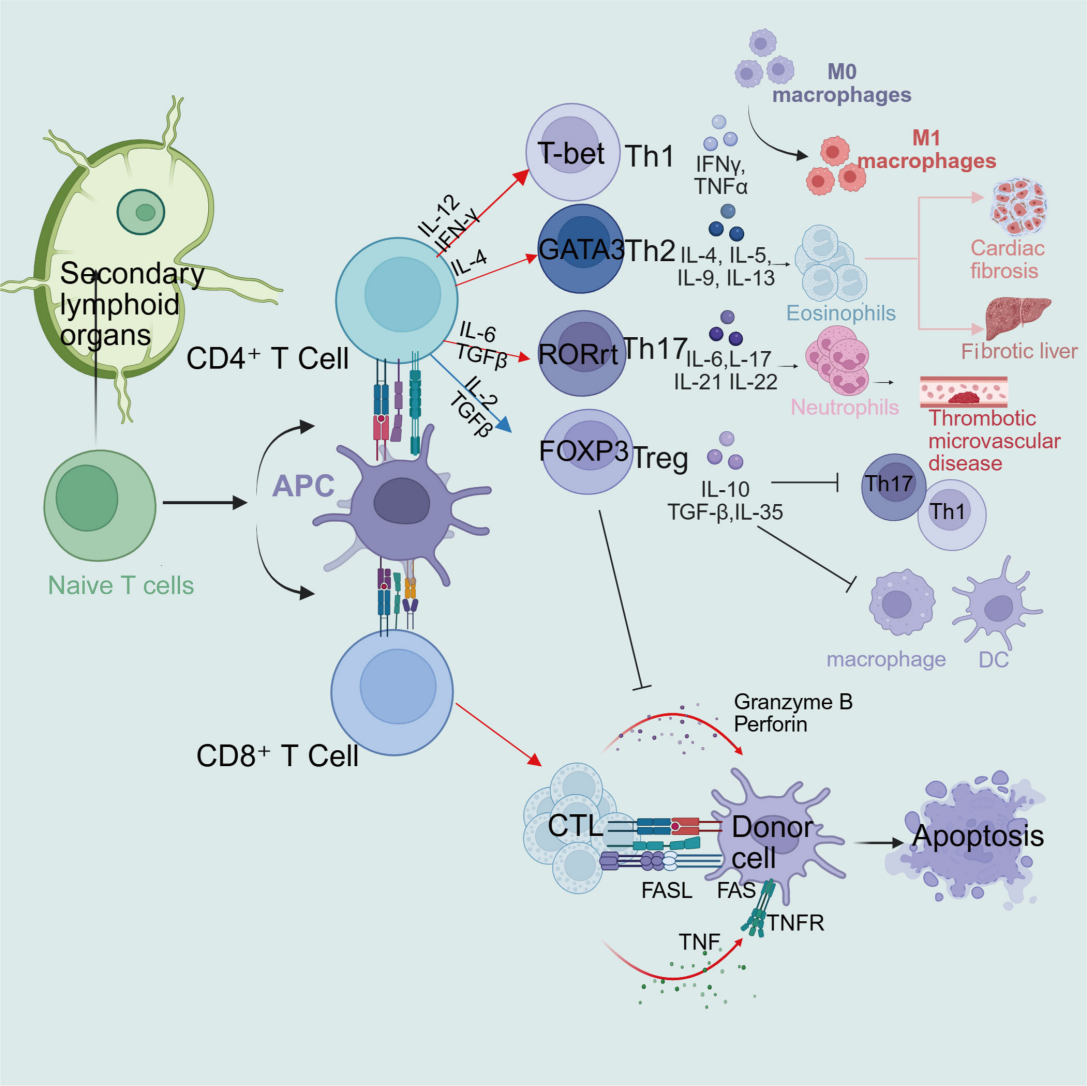
Different T cell subsets secrete distinct cytokines and perform specialized functions. In secondary lymphoid organs, naive T cells are activated by APCs and differentiate into distinct subsets: CD4^+^ T cells recognize MHC class II-peptide complexes through their TCRs and differentiate into Th1, Th2, Th17, or Tregs via distinct pathways, with each subset secreting characteristic cytokines to perform specialized functions. Th1, Th2, and Th17 cells primarily promote graft rejection, while Tregs induce immune tolerance; meanwhile, CD8^+^ T cells recognize MHC class I-peptide complexes and differentiate into CTLs that mediate direct killing through the perforin-granzyme pathway, the Fas/FasL pathway, and TNF-α/TNFR pathway. Created in https://BioRender.com.

CD4^+^ T cells differentiate into distinct effector subsets upon recognizing donor MHC class II antigens under the influence of local cytokine microenvironments. Undifferentiated CD4^+^ T cells can develop into various helper T cell subsets, including Th1, Th2, Th17, Th22, Th9, Tfh, and Tregs, through TCR activation in response to specific cytokine milieus (**Figure 5**). These subsets are primarily classified according to their characteristic cytokine secretion profiles and biological functions (46, 47). Here we mainly discuss Th1, Th2, Th17, and Tregs.

### Th1

Th1 cells, as a crucial effector subset of CD4^+^ T cells, play a central pro-inflammatory and pro-rejection role in organ transplant immunity (48).These cells primarily mediate ACR and chronic allograft dysfunction through the secretion of key cytokines including IFN-γ, TNF-α, and IL-2, which activate various immune cells (64). During acute rejection, Th1 cells exert their pathogenic effects through multiple mechanisms. First, IFN-γ secretion significantly upregulates the expression of MHC class I and II molecules on graft cells, enhancing donor antigen presentation efficiency and making the graft more susceptible to host immune recognition and attack (49). Second, IFN-γ activates macrophages and promotes their polarization toward the pro-inflammatory M1 phenotype. Activated M1 macrophages release large quantities of reactive oxygen species, nitric oxide, and pro-inflammatory cytokines, directly damaging graft tissues (50). Th1 cells can also infiltrate graft vascular endothelium, directly inducing endothelial cell apoptosis through FasL pathway while upregulating vascular adhesion molecules to promote inflammatory cell infiltration (51). Furthermore, IL-2 secreted by Th1 cells promotes the proliferation and activation of CD8^+^ CTLs, enhancing their ability to kill graft cells (52).

During chronic rejection, Th1 cells primarily contribute to progressive graft dysfunction by promoting vasculopathy and interstitial fibrosis. IFN-γ drives transplant arteriosclerosis by stimulating vascular smooth muscle cell proliferation, migration, and extracellular matrix deposition, which in turn activates the nuclear factor-κB pathway and upregulates pro-inflammatory factors and adhesion molecules, thereby exacerbating vascular endothelial injury (53–55). In terms of interstitial fibrosis, cytokines secreted by Th1 cells activate fibroblasts and promote the synthesis and deposition of collagen (particularly types I and III), ultimately leading to progressive organ dysfunction. Cardiac allograft vasculopathy, the leading cause of long-term mortality in heart transplant recipients, is pathologically characterized by abnormal coronary intimal hyperplasia and progressive luminal stenosis. Studies have confirmed that persistent abnormal activation of T cells serves as the central mechanism driving disease progression. Within the chronic inflammatory microenvironment, the Th1 cell subset activates vascular endothelial cells through IFN-γ secretion, not only promoting monocyte adhesion and infiltration but also directly inducing intimal hyperplastic responses. Concurrently, Th17 cells significantly stimulate vascular smooth muscle cell proliferation via IL-17 release, further exacerbating luminal stenosis (53, 56). Notably, during cardiac allograft fibrosis, local infiltration of Th1 cells activates cardiac fibroblasts and promotes their differentiation into myofibroblasts through an integrin α4-dependent mechanism. Moreover, Th1 cells induce aberrantly high expression of TGF-β in myofibroblasts, ultimately leading to pathological deposition of fibrotic extracellular matrix(ECM) in myocardial tissue and consequent maladaptive structural remodeling (53). Th1 cells do not function in isolation but rather participate in complex interaction networks with other immune cells (57). For instance, IFN-γ secreted by Th1 cells promotes antibody class switching in B cells, leading to production of complement-fixing IgG2a and IgG3 subclasses (58). These antibodies can exacerbate graft damage through both antibody-dependent cell-mediated cytotoxicity and complement activation pathways (23, 59). Additionally, Th1 cells can activate NK cells through IL-2 secretion, thereby enhancing their cytotoxic activity against graft cells.

### Th2

In organ transplantation, Th2 cells and their associated immune responses exhibit complex and dual roles. Primarily through the secretion of cytokines, including IL-4, IL-5, IL-10, and IL-13, Th2 cells participate in immune regulation with effects that may both suppress acute rejection and promote chronic rejection and graft fibrosis (60). On one hand, Th2 cell responses can inhibit the activation of Th1 cells and Th17 cells, while IL-4 and IL-10 may further suppress pro-inflammatory cytokine production (61), thereby attenuating acute cellular rejection. Under specific conditions, Th2 cells may also enhance Treg function to promote transplant tolerance (62).

On the other hand, Th2 cells may also promote collagen deposition and tissue fibrosis—such as interstitial fibrosis in kidney transplants or bronchiolitis obliterans in lung transplants—through activation of M2 macrophages and fibroblasts (61). Additionally, Th2 cells facilitate B cell production of DSAs, contributing to AMR, which represents a key mechanism of chronic allograft dysfunction (23, 63). Clinical studies demonstrate that Th2-associated cytokine levels correlate with distinct transplant outcomes: for instance, IL-10 may predict favorable long-term prognosis, whereas elevated IL-13 levels are often associated with chronic graft dysfunction (64, 65). Collectively, the role of Th2 cells in transplant immunity exhibits spatiotemporal dependency, with their ultimate effects determined by the synergistic interplay of cytokine microenvironments, transplanted organ type, and immunosuppressive regimens.

### Th17

The differentiation of Th17 cells depends on the STAT3-RORγt signaling axis. Their secreted IL-17 induces chemokine production that recruits neutrophils to the graft (66, 67). These neutrophils release reactive oxygen species (68), proteolytic enzymes (69), and neutrophil extracellular traps(NETs). NETs provide a scaffold that promotes platelet aggregation and fibrin deposition, leading to thrombotic microangiopathy, which directly damages vascular endothelium and parenchymal cells. Furthermore, NETs stimulate Th17 cell expansion and enhance IL-17 secretion, creating a pro-inflammatory feedback loop (70–72).

Th17 cells exhibit a dual functional nature: while capable of pro-inflammatory effects, they may also exert protective roles in transplanted organs. IL-17 can provide protection by maintaining epithelial barrier integrity, inducing antimicrobial peptide expression, and regulating tissue repair, thereby contributing to infection prevention and graft tolerance (73). The intestinal microenvironment harbors two functionally distinct Th17 subsets with divergent metabolic pathways and inflammatory profiles, homeostatic Th17 cells that rely on fatty acid oxidation metabolism, display weak inflammatory properties, and primarily mediate barrier defense; and pathogenic Th17 cells that are activated through glycolytic metabolism, highly express IL-17A and other inflammatory factors, and drive robust inflammatory responses (73, 74). Deteix and his research team discovered that Th17 cells can stimulate the formation of tertiary lymphoid structures and promote the aggregation of inflammatory effector molecules in transplanted tissues, leading to the development of ectopic germinal centers (75, 76). IL-17A expression has been associated with allograft dysfunction and rejection (75, 77). In MHC-mismatched mouse kidney transplantation models, researchers led by Kwan found that IL17A gene deficiency significantly attenuated transplant rejection and markedly prolonged graft survival time (75, 78). Furthermore, IL-17A increases vascular permeability by downregulating tight junction proteins (such as ZO-1 and occludin), thereby promoting edema and microthrombus formation (79).

In addition to secreting IL-17, Th17 cells can co-express IL-21, IL-22 (46), TNF, granulocyte-macrophage colony-stimulating factor, chemokines CXCL1, CXCL2, and CXCL8, as well as CCR6 and CCL20 (80). Research by Alexander et al. demonstrated that CCR6+ Th17 cells producing both IL-17 and TNFα play a pivotal role in intestinal transplant rejection by inducing potent pro-inflammatory Th17 responses (81).

Furthermore, multiple cytokines, including IL-6, IL-1β, IL-21, IL-23 and TGF-β, collectively regulate the differentiation of naive CD4^+^ T cells into Th17 cells (69, 82–84). Chronic antibody-mediated rejection(CAAMR) represents a serious clinical challenge in kidney transplantation, leading to graft function loss in approximately one-quarter of cases. Recent studies have demonstrated that inhibition of IL-6 signaling can significantly improve clinical outcomes in CAAMR patients (85, 86). In a study investigating the IL-6 antibody clazakizumab for treating late antibody-mediated rejection(ABMR) in kidney transplantation, Konstantin’s team found that renal transplant recipients treated with clazakizumab showed slower postoperative decline in estimated glomerular filtration rate, early reduction in DSA levels, decreased C4d scores, and resolution of ABMR activity in some patients. These results demonstrate that the IL-6 antibody clazakizumab plays a crucial therapeutic role in managing late ABMR following kidney transplantation (87).

### The direct cytotoxic effects of CD8^+^ CTLs

CD8^+^ CTLs mediate graft injury through multiple mechanisms, exerting complex and profound effects that influence long-term graft survival. Following organ transplantation, the foreign MHC class I molecules expressed by the donor organ are taken up and processed by host APCs, which activate host CD8^+^ T cells through cross-presentation pathways, leading to their differentiation into effector CTLs (5, 88). These activated CTLs recognize MHC class I-peptide complexes on donor cell surfaces via TCR engagement, primarily exerting direct cytotoxic effects through three pathways: the perforin-granzyme pathway, Fas/FasL pathway, and TNF-α/TNFR pathway. The perforin-granzyme pathway represents the predominant cytotoxic mechanism. CTL-derived perforin forms pores in target cell membranes, enabling granzyme B entry to directly damage transplanted organ parenchyma and activate caspase cascades that induce target cell apoptosis. This pathway serves as the principal means by which CTLs kill graft cells (5, 8). The Fas/FasL pathway induces apoptosis through death receptor signaling. CTLs express FasL on their surface, which binds to Fas(CD95) on target cells, activating the death receptor signaling pathway and leading to caspase-8 activation and subsequent apoptosis (89). This mechanism plays a significant role in rejection of transplanted organs such as liver and heart. In the TNF-α/TNFR pathway, CTLs secrete TNF-α, which binds to TNFR on target cell surfaces, inducing either apoptotic or necrotic cell death. Other members of the TNF/TNFR family also play roles in promoting primary CD8^+^ T cell responses (90). Research by Senji et al. demonstrated that TNF-α not only stimulates CTL proliferation by activating DCs, but also directly enhances CTL proliferation through its effects on CTLs themselves (91). CD8^+^ CTLs can complete the differentiation process in the absence of CD4^+^ T cell help and participate in the occurrence of transplant rejection through cytotoxic effects. This pathological process has been found to fully require the TNF-α/TNFR signaling pathway (92). Additionally, TNF-α can promote inflammatory responses, exacerbating graft injury (93).

These cytotoxic mechanisms act synergistically, leading to extensive damage to the parenchymal cells of the graft. In acute cellular rejection ACR, this manifests as interstitial lymphocyte infiltration, parenchymal cell apoptosis, and localized tissue necrosis. CTLs attack renal tubular epithelial cells, resulting in tubulitis and impaired renal function. Additionally, CTL-mediated hepatocyte necrosis can, in severe cases, lead to liver graft failure. In liver transplant recipients with well-functioning grafts, a rapid decline in donor-specific CD8^+^ T cells has been observed, indicating T cell inactivation and the development of tolerance (94, 95). Furthermore, in hematopoietic stem cell transplantation, donor-derived CD8^+^ CTLs may also attack host tissues, leading to GVHD (96, 97).

CD4^+^ T cell subsets synergize with CD8^+^ CTLs, drive acute inflammatory injury and chronic pathologies through a complex cytokine network and direct cytotoxic mechanisms. This interplay forms the pivotal immune response axis that determines the fate of transplanted organs, providing a theoretical foundation for targeted interventions. Although our understanding of the roles of T helper cell subsets and CTLs in transplant rejection has deepened, the intricate regulatory networks, spatiotemporal dynamics, and individual heterogeneity of these cells remain key challenges.

### Treg and the establishment of immune tolerance

Tregs play a central role in the establishment and maintenance of immune tolerance. Through multiple mechanisms, they suppress excessive immune responses, thereby maintaining the body’s tolerance to self-antigens and exerting crucial regulatory functions in transplantation immunity, autoimmune diseases, and chronic infections (10). The main subsets of Tregs include thymus-derived natural Tregs(nTregs) and peripherally induced Tregs(iTregs) (30), both of which highly depend on the expression of the transcription factor Foxp3 to maintain their phenotype and function (10). During thymic development, nTregs acquire immunosuppressive capabilities through medium-affinity TCR interactions with self-antigen-MHC complexes, coupled with cytokine stimulation that activates Foxp3 expression (98). In contrast, iTregs are peripherally induced from conventional CD4^+^ T cells under anti-inflammatory factors such as TGF-β (99), retinoic acid and low-dose antigen stimulation (100). Mature Tregs establish immune tolerance through multiple mechanisms: firstly, Tregs can suppress effector T cell activation via direct cell contact-dependent mechanisms (35, 37), such as competitively binding CD80/CD86 molecules on APCs through high CTLA-4 expression to block costimulatory signals (101, 102); secondly, Tregs secrete inhibitory cytokines, including IL-10, TGF-β, and IL-35 to create a local immunosuppressive microenvironment; additionally, Tregs can directly kill activated effector T cells and APCs through granzyme and perforin pathways, or inhibit proliferation and function of neighboring immune cells via metabolic interference, such as IL-2 consumption and adenosine release (34, 36, 103).

In the context of organ transplantation, Tregs play a crucial role in establishing transplant tolerance. Animal model studies have demonstrated that adoptive transfer of Tregs significantly prolongs graft survival, and this protective effect depends on Treg-mediated suppression of donor-specific effector T cells (particularly CD8^+^ CTLs) (102, 104). Tregs migrate to the graft site where they inhibit dendritic cell maturation and antigen-presenting function (105), block effector T cell activation and infiltration, and promote the polarization of tissue-reparative macrophages.

Stable expression of Foxp3 enables Tregs to maintain immune homeostasis and prevent autoimmunity (106, 107). Clinical observations reveal that the quantity of Foxp3^+^ Tregs in peripheral blood or grafts post-transplantation positively correlates with long-term graft survival. As a key lineage-defining transcription factor, Foxp3 exhibits dual regulatory functions: it activates the immunosuppressive gene expression program in Tregs while simultaneously suppressing the expression of characteristic functional genes in effector T cells. Research demonstrates that Treg functional deficiencies (including abnormal Foxp3 expression or insufficient cell numbers) can trigger acute and fatal systemic autoimmune responses (98).

**Figure 6:**
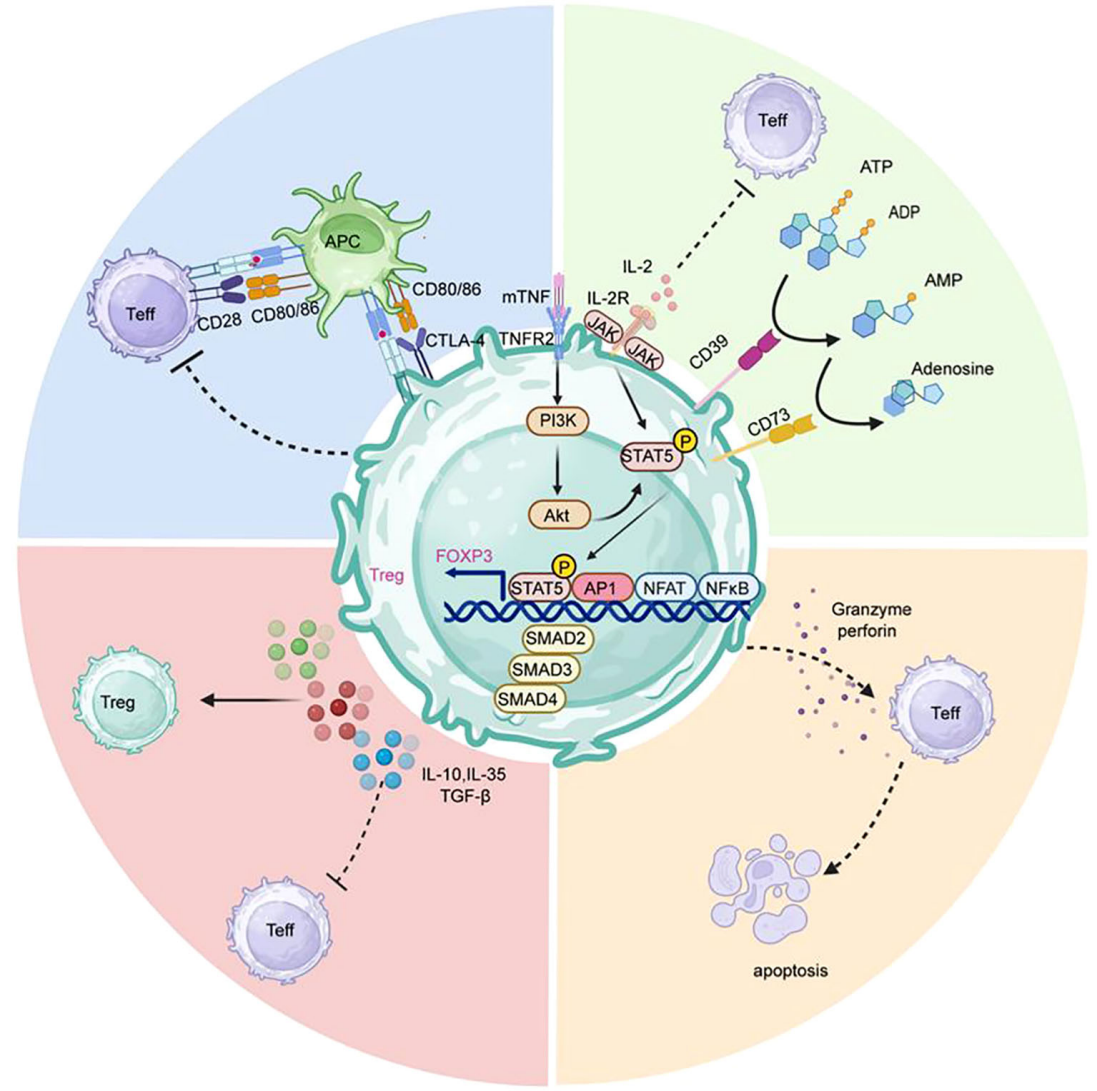
How Tregs Mediate Immune Tolerance. Within Tregs, the IL-2/IL-2R signaling axis dominates the differentiation process by inducing phosphorylation and activation of STAT5. While the binding of mTNF to TNFR2 activates the PI3K-Akt signaling pathway further promoting STAT5 phosphorylation and enhancing Foxp3 transcriptional expression. These signaling pathways collectively regulate Treg development and function. Tregs suppress effector T cell activation through multiple mechanisms: competitively binding to CD80/CD86 molecules on APCs via high CTLA-4 expression to block costimulatory signals; by secreting inhibitory cytokines such as IL-10, TGF-β, and IL-35 to create a local immunosuppressive microenvironment that simultaneously suppresses Teff cells and promotes Treg proliferation; through direct killing of activated effector T cells and APCs via the granzyme-perforin pathway; and by metabolic interference, including IL-2 consumption and adenosine release to inhibit neighboring Teff cell proliferation and function. Created in https://BioRender.com.

However, the role of Tregs in transplant tolerance faces significant challenges. Under inflammatory conditions with elevated cytokine levels, some Tregs may undergo functional conversion, transforming from immunosuppressive cells into pro-inflammatory effector cells (11). Conversely, excessive Treg activity can lead to immunodeficiency, increasing risks of infections and malignancies (**Figure 6**) (108). Therefore, current research focuses on developing strategies for targeted Treg modulation, or enhancing Treg stability and function through epigenetic modifications.

### Heterogeneity of T cell responses in different organ transplants and immunosuppressive strategies

The heterogeneity of T cell responses in different organ transplants is manifested not only in their overall intensity but more profoundly in the recruitment, differentiation, and functional preferences of T cell subsets (3). These differences directly determine the pathological features of rejection and impose organ-specific requirements on the selection of treatment strategies and the development of novel therapies.

The liver, often referred to as an “immunologically privileged organ,” exhibits relatively favorable tolerance, which is attributed not only to its unique structure but also to distinct T cell subset dynamics. The graft is often enriched with Tregs, which create a potent local immunosuppressive microenvironment through the secretion of IL-10 and TGF-β (109, 110). Liver-resident antigen-presenting cells (such as liver sinusoidal endothelial cells and Kupffer cells) tend to induce T cell anergy or apoptosis rather than activation (111, 112). Therefore, the clinical protocol for liver transplantation emphasizes early and rapid drug tapering, often exploring regimens that combine low-dose calcineurin inhibitors(CNIs) with mammalian targets of rapamycin(mTOR) inhibitors to promote Treg generation and suppress proliferation of allogeneic effector T cells. Rapamycin inhibits the proliferation and differentiation of effector T cells by inhibiting mTORC1 activity, and when combined with low-dose IL-2, it significantly improves Foxp3 stability and Treg abundance (113–115), thereby consolidating the liver’s inherent tolerogenic propensity (116, 117). In addition to standard immunosuppression, treatment strategies for liver transplantation are actively exploring low-dose IL-2 therapy to selectively expand Tregs or the adoptive transfer of *in vitro*-expanded Tregs to reinforce the liver’s inherent tolerogenic propensity (118).

In contrast, lung and intestinal transplants face higher risks. These organs are continuously exposed to external antigens, and their mucosal immune systems are in a state of baseline activation (119), making them more prone to polarizing toward effector subsets such as Th2, Th17, and Th22 post-transplantation. These cells, through the secretion of cytokines like IL-17 and IL-22, potently recruit neutrophils and drive epithelial barrier damage and fibrosis (120), serving as key drivers of bronchiolitis obliterans syndrome (121) and chronic enteritis. Traditional broad-spectrum T cell inhibitors often have limited efficacy. Novel therapeutic options are thus focusing on targeting this specific pathway, for example, using IL-6R or IL-23 monoclonal antibodies to inhibit Th17 differentiation, or exploring the modulation of local microbiota to indirectly alter T cell polarization (122).

For heart and kidney transplants, Th1 and effector CD8^+^ T cells are the core forces mediating acute vascular rejection and interstitial inflammation (5). The high immunogenicity of cardiac graft vascular endothelial cells and the rich capillary network in the kidneys provide targets for these effector cells. The clinical protocol requires potent suppression of effector T cells. In kidney transplantation, a triple-drug regimen consisting of CNIs, corticosteroids, and antiproliferative agents is often used for early and potent rejection prophylaxis (123, 124). Novel precision strategies focus on costimulation blockers, which more precisely intervene in full T cell activation by selectively blocking the CD28-B7 signal (125, 126). These agents have demonstrated superior long-term renal function protection compared to conventional immunosuppressive regimens in kidney transplantation. Furthermore, for patients with end-stage heart failure, research is exploring the use of chimeric antigen receptor T cells (CAR-T) to target and eliminate activated fibroblasts infiltrating the transplanted heart (127, 128), thereby counteracting T cell-mediated interstitial fibrosis.

## Discussion

This review systematically elucidates the central role of T cells in solid organ transplant immunity, revealing their dual functions in mediating rejection and maintaining immune tolerance. Studies demonstrate that T cells recognize donor antigens through direct, indirect, and semi-direct pathways, and differentiate into functionally diverse effector subsets that collectively determine graft fate (46, 47, 129).

Although current immunosuppressive regimens, primarily based on calcineurin inhibitors, have significantly reduced the incidence of acute rejection, their non-specific suppression leads to metabolic side effects, coupled with limited efficacy against chronic rejection and antibody-mediated rejection, constituting major bottlenecks in clinical practice (3, 130). In recent years, strategies such as costimulation blockade, cytokine-targeted therapy, and Treg expansion have demonstrated potential for shifting toward precise immune modulation (131). However, several fundamental challenges and controversies remain in this field. The balance between effector and regulatory T cell functions is highly dynamic and microenvironment-dependent, and the issue of Treg functional instability—or even conversion to a pathogenic phenotype under inflammatory conditions—remains unresolved. Discrepancies between preclinical models and the human immune system, along with significant immunological heterogeneity among patients, complicate the translation of findings from animal studies to clinical applications (132, 133). More critically, existing research predominantly focuses on T cells themselves, with insufficient attention to their collaborative networks with B cells and innate immune cells, thereby limiting a holistic understanding of rejection mechanisms and the comprehensive design of therapeutic strategies (134, 135).

In the future, we need to utilize new technologies such as single-cell sequencing and spatial transcriptomics to deeply analyze the spatiotemporal dynamic landscape of immune cell interactions after transplantation and identify reliable biomarkers for predicting rejection or tolerance. We must also focus on developing spatiotemporally specific delivery systems to achieve localized immune modulation and reduce systemic impact (136, 137). At the same time, it is crucial to actively explore combination therapies to stably induce tolerance, implement individualized immunosuppressive regimens (133). Additionally, pioneering approaches such as adoptive transfer of engineered Tregs (138), constructing specifically regulated cells using gene-editing technologies (139), CAR-T principles, and *in situ* cell therapy pave new pathways toward achieving truly personalized and long-lasting immune tolerance (140).
